# Plasticity of Horizontal Connections at a Functional Border in Adult Rat Somatosensory Cortex

**DOI:** 10.1155/2009/294192

**Published:** 2010-03-03

**Authors:** Sally A. Marik, Peter W. Hickmott

**Affiliations:** ^1^Laboratory of Neurobiology, The Rockefeller University, 1230 York Avenue, New York, NY 10065, USA; ^2^Departments of Psychology and Interdepartmental Neuroscience Program, University of California, Riverside CA 92521-0426, USA

## Abstract

Horizontal connections in superficial cortical layers integrate information across sensory maps by connecting related functional columns. It has been hypothesized that these connections mediate cortical reorganization via synaptic plasticity. However, it is not known if the horizontal connections from discontinuous cortical regions can undergo plasticity in the adult. Here we located the border between two discontinuous cortical representations in vivo and used either pairing or low-frequency stimulation to induce synaptic plasticity in the horizontal connections surrounding this border in vitro. Individual neurons revealed significant and diverse forms of synaptic plasticity for horizontal connections within a continuous representation and discontinuous representations. Interestingly, both enhancement and depression were observed following both plasticity paradigms. Furthermore, plasticity was not restricted by the border's presence. Depolarization in the absence of synaptic stimulation also produced synaptic plasticity, but with different characteristics. These experiments suggest that plasticity of horizontal connections may mediate functional reorganization.

## 1. Introduction

Throughout the brain, there are functionallyorganized regions. Of particular interest are sensory maps, which produce orderly representations of incoming sensory stimuli. One important feature of such maps is that they are discontinuous, divided into discrete functional subregions. For example, in primary somatosensory cortex (S1) the map is divided into regions activated by a particular part of the body surface. Theseregions are separated from each other by distinct physiological borders. Thus, a border represents a constraint on the spread of excitation through the cortical circuit. The balance of excitation and inhibition, along with the anatomical spread of connections (axons and dendrites), will determine the overall spread of excitation. Horizontal connections within superficial cortex are known to participate in integrating information across cortical regions, primarily by connecting regions with similar response properties [1]. 

Cortical circuits are capable of undergoing experience dependent modification throughout life. One manifestation of this adaptation is the remapping of cortical topography following sensory loss. In the somatosensory cortex, peripheral nerve damage leads to shrinkage in the corresponding deprived cortical representation and an expansion of the adjacent cortical representations into the deprived cortical area. This remapping of function occurs immediately and continues to progress in the subsequent weeks and months [2–8]. While these connections play a role in sensory integration in the normal cortex, they can be modulated for the purpose of topographic remapping and by a process of axonal sprouting and synaptogenesis [7].

A significant aspect of circuitry that undergoes cortical reorganization in somatosensory cortex is the border region between the deprived and nondeprived cortical regions. Both the morphological and functional aspects of the circuitry surrounding a border are altered by the border's presence [9–12]. Neurons located in close proximity of a border have both dendritic [11, 13] and axonal [9, 14] biases towards the center of their home column. Additionally, the presence of a border limits the spread of excitation and inhibition to adjacent representations [10, 12, 14]. This phenomenon may be partially explained by the fewer axons projecting into discontinuous representations [9]. Interestingly, the morphological and functional biases of the original border relocate with the reorganized border following sensory loss [13, 15–17].

A key feature of cortical reorganization following sensory loss is the breakdown and/or shifting of the normal border and the creation of a new border that is located within the deprived cortical representation. This shift of the border occurs rapidly following sensory loss [3, 16, 18]. Layer II/III horizontal connections are the first to undergo reorganization following sensory loss [19]. One proposed mechanism for this phenomenon is a change in synaptic efficacy of horizontal connections. Long-term potentiation (LTP) and long-term depression (LTD), both well-studied forms of synaptic plasticity, are ideal candidates for this immediate phase of cortical reorganization because they are both quick to induce and their effects are long lasting [20]. Pairing postsynaptic depolarization with stimulation is a reliable way of inducing LTP in many circuits of the brain [21–32] and low frequency stimulation (LFS) reliably yields LTD [33–40]. LTP and LTD have been extensively studied at isolated excitatory synapses; however little is known about coordinated changes in excitation and inhibition in individual neurons resulting from pairing or LFS. Furthermore, LTP and LTD have been studied mostly in juvenile brain circuits, and some circuits lose their plastic abilities with age [41].

It is unknown whether the horizontal connections in adult layer II/III somatosensory cortex are capable of LTP and/or LTD when a border is present and the characteristics of such plasticity, particularly whether it is pathway specific. To explore the synaptic plasticity capabilities of superficial horizontal connections we combined an in vivo/in vitro approach to determine if horizontal axons traversing through continuous (border absent) or discontinuous (border present) regions of representations are capable of LTP and/or LTD and if they differ in their ability to undergo synaptic plasticity. Postsynaptic potentials (PSPs) that contained both excitation and inhibition were examined to determine possible coordinated changes in excitation and inhibition. Since a border represents a discontinuity in the circuit, the patterns of activation of connections that cross over the border will differ from those that are within a continuous representation. We therefore hypothesized that the ability of synaptic responses to undergo LTP and LTD will differ in connections that cross the border versus those that do not.

## 2. Materials and Methods

All animal procedures followed NIH institutional guidelines and were approved by the University of California at Riverside IACUC. Adult female Sprague Dawley rats (280–350 g; age 3 months or older) were anesthetized using pentobarbital (administered intraperitoneally, 50 mg/kg) until areflexic and placed in a stereotaxic frame. Temperature was maintained at 38 degrees C with a heating pad and rectal thermometer. Lidocaine (2%) was administered subcutaneously to pressure points and at places of incision. All chemicals were purchased from Sigma-Aldrich unless otherwise stated.

### 2.1. In Vivo Surgery

To determine the location of the lower jaw/forepaw border, the region of S1 at the border was physiologically mapped [10]. A small incision was made slightly off center from the midline, the skin and temporalis muscle were reflected back, and a craniotomy made over the region of interest in S1. A cisternal drain was performed to reduce edema of the cortex while recordings were performed. Multiunit responses were recorded with a custom made carbon fiber electrode (10 *μ*m fiber diameter). Carbon fibers were made by placing the carbon fiber into a glass pipette, and pulled on a Flaming/Brown puller (Sutter Instruments) so the carbon fiber extended beyond the tip of the glass pipette, and the glass pipette was filled with 3 M NaCl. Recordings were taken between 600 and 700 *μ*m below the cortical surface, in response to stimulation of the periphery (forepaw and lower jaw) with a glass rod. Responses were amplified 10,000 times (A-M Systems Microelectrode A.C. Amplifier) and then fed into an audio monitor (Grass AM10). Blood vessels were used to determine the location of the electrode in the cortex and to record the responses at each penetration (paw, jaw, or both). An image of the exposed cortex was taken using a Pixera digital camera (Pixera Corp). Responses were recorded on the image using Canvas 5.0 (Deneba Systems Inc.) and stored on a Macintosh G4 computer. Electrode penetrations were spaced 50 *μ*m or less from each other in the medial-lateral dimension to precisely determine the location of the border. Three to four of these closely spaced series of penetrations were acquired at 500 *μ*m intervals (in the rostral/caudal dimension). After the mapping was complete, 3-4 sites with strong responses for both forepaw and lower jaw stimulation (i.e., at the border) were marked by coating the recording electrode with 2% DiI dissolved in ethanol [42]. Then, the electrode was advanced into the cortex at border sites for 2 minutes to allow DiI crystals to be deposited.

### 2.2. In Vitro Preparation

**Figure 1:**
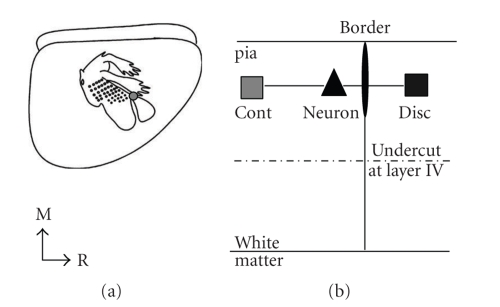
The in vivo and in vitro preparations. (a) Lateral view of the rat brain with a schematic map of S1 overlaid. The gray circle depicts one location of forepaw/lower jaw border. Recordings were made in vivo to determine the location of the border and the border was marked with DiI. (b) Schematic of in vitro slice recording. The top solid line depicts pia and the bottom solid line depicts white matter, the border is the vertical thin line, and the border marked with DiI is the thin oval. The recording electrode recorded from a single neuron (triangle) approximately 100 *μ*m from the marked border (black oval) and two stimulating electrodes were placed at the same depth from the pia at 300 *μ*m from the tip of the recording electrode for continuous representation (gray square) and discontinuous representation stimulation (black square). Layer four was undercut (dashed line) in order to isolate the horizontal connections.

After mapping and marking the border, the animal was decapitated and the brain excised. Coronal slices (400 *μ*m thick) were cut on a vibratome (Leica VT1000s) and sections were maintained in bicarbonate buffer (in mM: NaCl, 119; KCl, 2.5; NaH_2_PO_4_, 1.25; MgSO_4_, 1.3; CaCl_2_, 2.5; NaHCO_3_, 26.2; glucose, 11; saturated with 95%O_2_/5%CO_2_) for intracellular recording. Slices were undercut at layer 4 (500–700 *μ*m from the cortical surface) to isolate supragranular responses. The forepaw/lower jaw border marked with DiI was detected using an epifluorescent microscope. Visible local landmarks, such as vasculature, were used to locate the DiI mark. Blind whole cell recordings were made approximately 100 *μ*m from the marked border and within layer II/III. Patch electrodes were pulled on a Flaming/Brown puller (Sutter Instruments), to a tip diameter of 1.5–2.5 *μ*m and filled with, in mM: KOH, 128; KCl, 7; EGTA, 0.1; HEPES, 10; Mg-ATP, 2; Na-GTP, 0.2; biocytin 0.3%–0.5%; pH 7.0–7.4 using D-gluconic acid; tip resistances were 3–8 MΩ. Stimulating electrodes were bipolar, parylene coated tungsten electrodes with tip separation of 50 *μ*m. Furthermore, one pole of the electrode was shorter by ~75 *μ*m; thus, when the active pole was placed in the tissue for stimulating, the other was slightly above the tissue. This configuration allowed for precise localization of the stimulating site. Stimulating electrodes were placed 300 *μ*m from the recording electrode on both sides and at the same distance from the cortical surface (Figure 1(b)). *Thus, one electrode stimulated fibers that crossed the border (discontinuous representation pathway) while the other stimulated fibers that were within the representation of the neuron that was being recorded from (continuous representation pathway).* These pathways have also been referred to as cross border (CB) and noncross border (NCB) [9–12, 15, 16]. All cells were recorded in current clamp mode and current was injected to keep the resting membrane potential at −70 mV, except during the pairing or depolarization protocol. Care was taken to prevent washout of cell, by using ATP and GTP in the filling solution, collecting only 5 minutes of baseline and initiating the plasticity paradigm within 10 minutes of recording from the cell. The pathways were stimulated at 0.1 Hz alternating between the two stimulating electrodes throughout the entire experiment. Stimulation intensities of the two sites were adjusted to result in a postsynaptic potential (PSP) of half the amplitude required to trigger a spike (~10 mV) and of approximately equal amplitude between the two pathways. In a subset of cells, smaller PSPs (3–5 mV) were used; since there were no significant differences seen in the results between the two groups, both small and larger amplitudes were pooled in analysis. Five minutes of baseline data were followed by either the LTP paradigm or the LTD paradigm. 

We are confident that this stimulation paradigm activated discrete populations of afferents for several reasons: The low intensity stimuli (<0.09 mA) yielded PSPs with amplitudes of 3–5 mV. Considering that single-fiber PSPs in these projections had a mean amplitude of ~0.7 mV [43], only a small number of fibers would be activated by these small stimuli. The low stimulus intensity yielded identical results to the higher intensity, indicating that there was no difference in the populations of afferents activated by higher and lower stimuli. Previous data from single [43] and multifiber [10, 12, 16] responses clearly demonstrate that the properties of PSPs differ considerably between discontinuous and continuous pathways. Thus, if there is overlap between the afferent populations, it is not sufficient to obscure differences between the pathways activated. Given this specificity in afferent stimulation, we refer to homosynaptic, heterosynaptic, and associative effects throughout this paper. Homosynaptic refers to changes in response that occur in the same pathway that was subject to pairing/LFS (i.e., continuous pathway pairing yielding continuous pathway change); heterosynaptic refers to changes in response that occur in the pathway that was not subject to pairing/LFS (i.e., continuous pathway pairing yielding discontinuous pathway change); associative refers to changes in response that occur in both pathways after pairing/LFS in one of the pathways (i.e., continuous pathway pairing yielding discontinuous and continuous pathway change).

### 2.3. LTP Paradigm

To induce LTP, the neuron was depolarized by current injection at a level where it fired ~10 spikes in the 200-milliseconds of depolarization. This depolarization was paired 30 times with the PSP resulting from stimulation of discontinuous representation and/or continuous representation pathways. The first spike led the PSP by 50 milliseconds. During pairing, the alternation of the location of stimulation at 0.1 Hz was maintained. In one set of experiments, both pathways were paired, In another set of experiments, only one pathway was paired. For these experiments the nonpaired pathway received the baseline stimulation during the pairing. After the pairing paradigm, PSPs were recorded for at least 20 minutes. In another group of cells, only the 200 millisecond depolarization was given. The current injected into the neuron was adjusted, so the neuron fired ~10 action potentials in the 200 millisecond, as in the pairing paradigm. However, no synaptic stimulation (including the 0.1 Hz baseline stimulation) was given for these neurons during the depolarization paradigm. 

The appropriate stimulation intensity of each pathway to elicit half the amplitude to spike or lower intensity remained constant for the duration of the recording of each cell. For all experiments (except when noted) PSPs were evoked by alternate stimulation of both horizontal pathways at 0.1 Hz. Stimulation intensities were adjusted so that PSPs were of approximately the same amplitude, and half the amplitude required to elicit a spike for both continuous representation and discontinuous representation pathways.

### 2.4. LTD Paradigm

After baseline recordings, LTD was elicited by low-frequency stimulation (LFS; 1 Hz for 900 pulses). LFS was applied to only one pathway (continuous representation or discontinuous representation pathway) due to the risk of washout of LTD because of the length of time necessary to apply LFS. LFS stimulation was not given at the same time to both pathways in order to avoid summation of the stimulation from the two pathways. During the presentation of LFS no stimulation was given to the other pathway. After LFS, the alternating 0.1 Hz stimulation resumed for post-LFS responses. Once LFS was given to a slice, it was discarded after the termination of that neuron's recording session.

### 2.5. Analysis

Recorded signals were amplified using an Axoclamp 2B amplifier (Axon Instruments), digitized at 10 kHz, and saved on a Macintosh G4 computer using Igor Pro (Wavemetrics, Inc.) data acquisition systems. Any cell whose input resistance changed by more than 15% over the course of the experiment was not included in the analysis. Amplitudes (from baseline to peak) of PSPs were analyzed off line. PSPs of five minute bins were averaged for baseline and postpairing. Additionally, postpairing averaged responses were compared to baseline responses to determine the magnitude of enhancement after the pairing. Slopes were determined for the initial rise of the PSP. This was done by finding the slope between the 20% and 80% points of the initial rise. Amplitudes and initial slopes were quantified by considering averaged baseline data 100%. Postpairing data were determined as a percentage by using the following formula: averaged post-PSP amplitude (or slope) divided by averaged pre-PSP amplitude (or slope) times 100. Any deviation over 15 percent of baseline that persisted 20 minutes postpairing or LFS was considered LTP, and any deviation under 15 percent of baseline that persisted beyond 20 minutes postpairing or LFS was considered LTD. Twenty minutes postpairing/LFS was chosen to maximize the number of neurons sampled, as it was difficult to hold cells for long periods of time (>35 minutes for the entire experiment). For cells that were held longer, there was no significant difference in PSP amplitude at 20 minutes and at 40 minutes (not shown); so the data at 20 minutes accurately represents long-term effects. Statistical significance was determined using one-way ANOVAs, followed by individual comparisons using a student's *t*-test. *P*-values of less than.05 were significant. Data are presented as means ± standard error of the mean (SEM).

During recording of most cells, biocytin was allowed to diffuse into the neuron and general morphology was later examined (data not shown) because experiments were done using blind whole cell patch recordings. The neurons recorded here were composed of layer II/III excitatory neurons. The neurons also displayed regular spiking behaviors of typical layer II/III cells [44] and had typical morphology of excitatory layer II/III pyramidal neurons.

## 3. Results

A total of 110 neurons from 71 animals were studied; the minimum duration of recording was 45 minutes. Based on the firing patterns of action potentials in these cells, 100% were regular spiking pyramidal cells. We analyzed resting membrane potentials, input resistance, distance between the border and the cell recorded, and distance between the pia and the cell. None of these parameters significantly differed between cells (Table 1). The mean initial PSP amplitude for continuous representation pathway stimulated pathways was 9.14 ± 0.004 mV, and for discontinuous representation pathways was 8.97 ± 0.004 mV. The values recorded here were similar to PSPs reported in [10]. PSPs consisted of monosynaptic and polysynaptic components as well as excitatory and inhibitory components [10]. In the absence of pairing, the amplitude and slope of the PSP were stable for over 60 minutes (*N* = 8, data not shown); therefore, the 0.1 Hz stimulation had no affect on the PSP response. 

It is important to note that inhibition was not blocked so that the PSPs consisted of coordinated EPSPs and IPSPs. Using this paradigm, we were able to view synaptic changes more similar to those that might occur in vivo. Given the short range of stimulation (~300 *μ*m), measurements of the PSP peak amplitude and rise time will be affected by both direct and indirect EPSPs and IPSPs. To ensure that the initial size of the PSP did not affect the ability to induce synaptic plasticity, some experiments were also done at lower amplitudes (4-5 mV). The initial amplitude of the PSP had no effect on the ability to induce LTP or the magnitude of enhancement. Since there was no effect of initial amplitude on the results, the data were pooled.

### 3.1. Single-Pathway Pairing: Population Analysis

**Figure 2:**
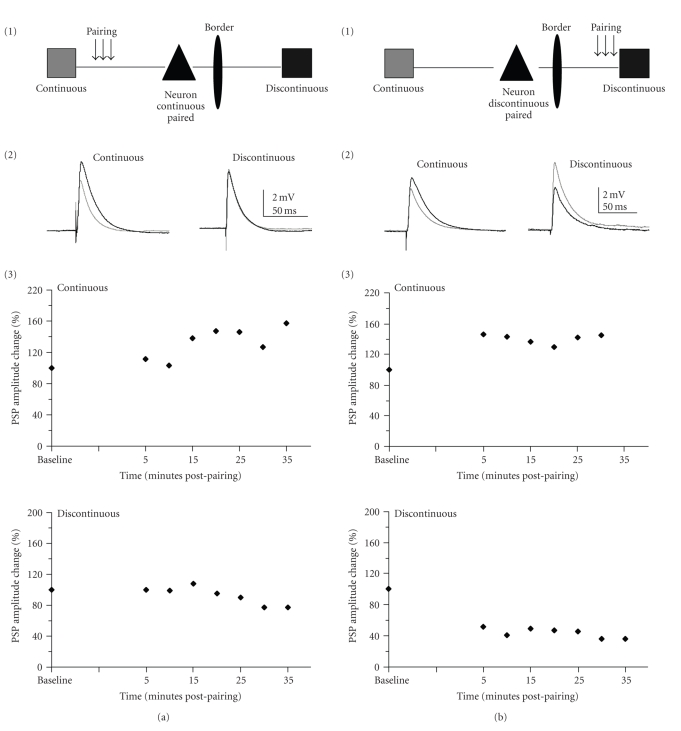
Changes in PSP amplitude generated by one pathway pairing. (a) Sixteen neurons underwent pairing of continuous representation connections only. (b) Fourteen neurons underwent pairing of discontinuous representation connections only. (1) Schematic of pathway pairing paradigm. (2) Example responses from a cell in which pairing of the indicated pathway induced homosynaptic (a) or homo- and heterosynaptic (b) enhancement of the pathways. Traces are averaged over five minutes. Grey traces are averaged PSPs from before pairing (baseline) and the black traces are averaged PSPs from 20 minutes postpairing. (3): % change of PSP amplitude over time for the cell shown in (2). PSP amplitude change was averaged over five minute intervals.

By using our in vivo/in vitro approach we were able to directly compare the potential for plasticity for horizontal connections crossing a functional border versus horizontal connections within a representation (Figure 1). We paired robust depolarization with stimulation of one of the two pathways to attempt to induce LTP (Figure 2). By pairing depolarization with only one of the two pathways in a subset of cells we were able to determine what types of plasticity were possible in these connections and what were the differences in plasticity outcomes depending on the pathway subject to pairing (*N* = 30). Pairing was applied to either the continuous (Figure 2(a)) or discontinuous (Figure 2(b)) pathway. There was no significant difference in the mean population PSP amplitude (Wilcoxon test; continuous *P* = .09; discontinuous *P* = .09) after pairing of either pathway. However, individual neurons did undergo plasticity for both the paired (homosynaptic plasticity) and unpaired (heterosynaptic plasticity) pathways (Figures 4(a) and 4(b); Table 2).

### 3.2. Dual Pathway Pairing: Population Analysis

**Figure 3:**
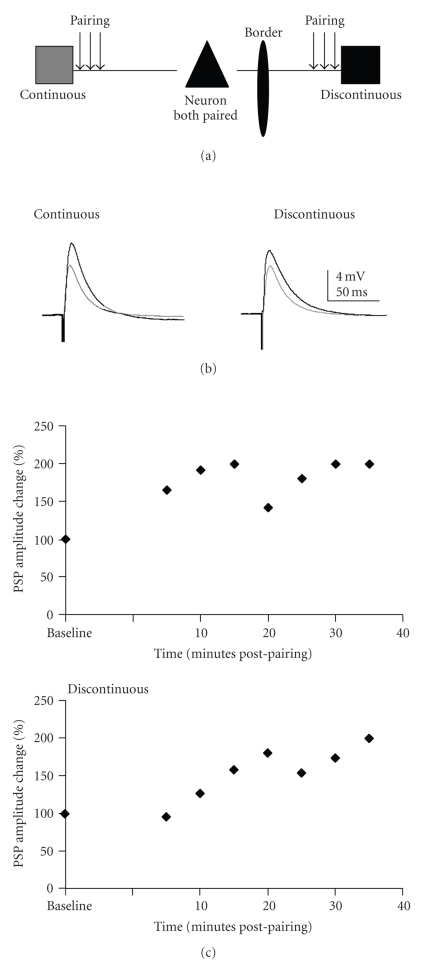
LTP induced by a pairing paradigm given to both continuous representation and discontinuous representation pathways. (a) Schematic drawing of the pairing protocol. Both pathways are paired alternately to induce synaptic plasticity. (b) Example of PSPs from a cell where both pathways underwent LTP. Gray traces are averaged baseline PSPs. Black traces are averaged traces 20 minutes postpairing. Traces are averaged over 5 minutes (15 traces) for both pre- and postpairing PSPs, (c) % change of PSP amplitude over time for the cell shown in 2. PSP amplitude change was averaged over five minute intervals.

**Figure 4:**
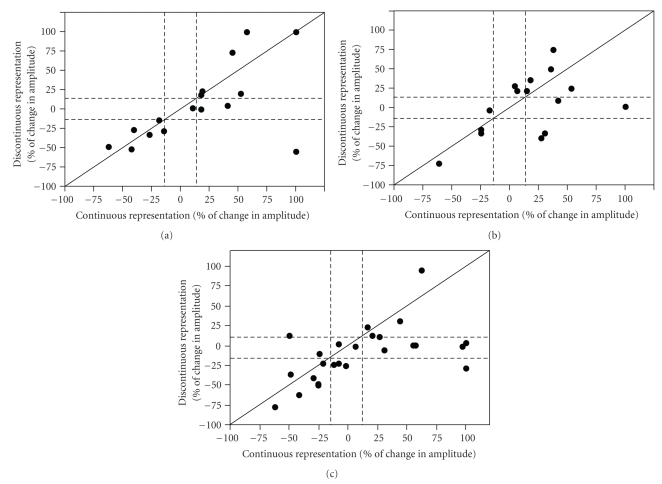
Individual neuron data. (a) Continuous pathway pairing. Change in PSP amplitude (expressed as percent of the baseline PSP amplitude) for continuous representation pathway plotted against discontinuous representation pathway for each cell that received continuous pathway pairing. Solid diagonal line depicts slope of one. Dashed lines depict 15% above and below no change (100%). (b) Discontinuous pathway pairing. Change in PSP amplitude (expressed as percent of the baseline PSP amplitude) for continuous representation pathway plotted against discontinuous representation pathway for each cell that received discontinuous representation pathway pairing. Conventions are as in (a). (c) Both pathway pairing. Change in PSP amplitude (expressed as percent of the baseline PSP amplitude) for continuous representation plotted against discontinuous representation pathway for each cell that received both pathway pairing. Conventions are as in (a).

In order to examine possible interactions between homosynaptic (input specific) and heterosynaptic (not input specific) plasticity, the pairing paradigm was presented to both pathways onto a single target neuron (Figure 3). There was not a significant difference in the mean amplitudes before and after pairing for either pathway (Figure 4(c); Wilcoxon test). However, the two pathways were significantly different in the percent change in PSP amplitude (Wilcoxon test; *P* = .01). This was due to a significant reduction in the mean percent change in the discontinuous pathway. Individual neurons exhibited a range of plasticity outcomes following dual pathway pairing (Figure 4(c); Table 2).

### 3.3. Categorization of Individual Changes

Figure 4 shows plots of the percent change in PSP amplitude for the discontinuous and continuous pathways resulting from the 3 pairing protocols discussed above. From these plots, it is clear that, even though there was no significant change in the overall *mean* amplitude, pairing of PSPs with depolarization produced various examples of potentiation or depression of the discontinuous or continuous representation pathway. Distinct populations of similarly-responding neurons are apparent in these plots. We first attempted to group the data based on correlation analysis with several independent variables. We found no correlation between the direction of plasticity and the following parameters: (1) the first inter-stimulus interval of the first two action potentials elicited from the depolarization in the pairing paradigm (*r* = −0.128 continuous paired; *r* = −0.469 discontinuous paired; *r* = −0.170 continuous in both paired; *r* = −0.122 discontinuous in both paired); (2) the time from the first action potential of the depolarization to the paired PSP (*r* = −0.491 continuous paired; *r* = 0.370 discontinuous paired; *r* = 0.062 continuous in both paired; *r* = 0.085 discontinuous in both paired); (3) the amplitude of the after hyperpolarization (AHP) following the pairing paradigm (*r* = 0.071 continuous paired; *r* = −0.121 discontinuous paired; *r* = −0.123 continuous in both paired; *r* = −0.185 discontinuous in both paired); or (4) the spiking pattern of the neuron (e.g., regular, fast spiking and bursting) (*r* = 0.473 continuous paired; *r* = 0.079 discontinuous paired; *r* = 0.176 continuous in both paired; *r* = 0.229 discontinuous in both paired). Therefore, we grouped the cells by their functional outcomes resulting from the pairing paradigm. Cells were functionally grouped by our standard +/−15 percent criterion for potentiation and depression. The majority of neurons showed a significant change in amplitude following pairing (>15%, dashed lines), indicated by their data points falling outside the dashed lines (Figure 4). The data from cells grouped by this criterion are shown in Table 2.

The direction of plasticity (enhancement, depression, or no change) was significantly different for the two pathways following both pathway pairing (chi-square, *P* < .001). This was not observed when only one pathway was paired (homosynaptic inputs, chi-square, 0.63; heterosynaptic inputs, chi-square, 0.37).

### 3.4. Location of Pairing Affects Plasticity Outcomes: Individual Cell Data

By examining the data using functional outcomes interesting features of layer II/III plasticity emerged (Figure 4, Table 2). For horizontal connections confined to a continuous representation, homosynaptic (in 25% of neurons) and heterosynaptic LTP (in 28.6%,) were both observed. However, when a horizontal pathway encompassed discontinuous representations, only homosynaptic LTP was observed (in 21.4% of neurons). Additionally, a percentage of neurons underwent depression of both pathways following pairing of the pathway from the continuous representation (37.5% of neurons) and discontinuous representation (21.4% of neurons). When both pathways were paired the discontinuous pathway only underwent plasticity when the continuous pathway underwent plasticity. Interestingly, the overall pattern of change for two-pathway pairing looked very similar to the pattern obtained by pairing of the continuous representation (Figures 4(a) and 4(c), Table 2). This suggests that when both pathways are paired the discontinuous representation does not contribute. 

For all three pairing paradigms, the continuous pathway was more likely to undergo potentiation (in 62.5%, 57.2%, and 44% of cases for pairing of the continuous, discontinuous and both pathways) than the discontinuous (in 37.5%, 50%, and 12% of cases for pairing of the continuous, discontinuous, and both pathways). The continuous pathway plasticity was also not input specific since LTP was induced when only the other pathway was paired (in 28.6% of cases). This was never observed for the discontinuous pathway.

### 3.5. LTP of Horizontal Connections Is NMDAR Dependent

**Figure 5:**
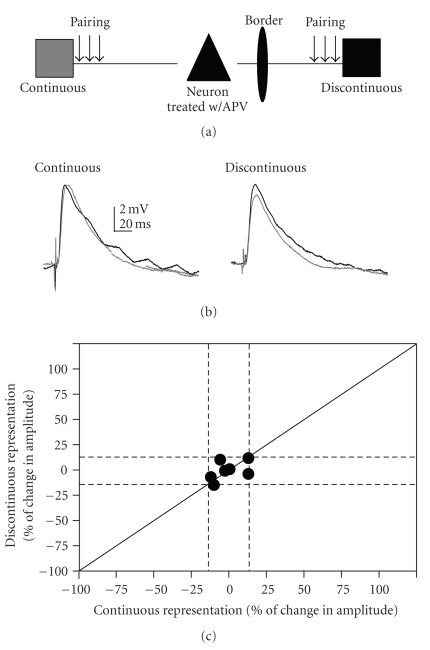
APV blocks pairing induced LTP. (a) Schematic of the two-pathway pairing paradigm. (b) Example responses from a cell that was treated with APV; gray traces are averaged baseline PSPs. Black traces are averaged traces 20 minutes postpairing. Traces are averaged over 5 minutes (15 traces) for both pre- and postpairing PSPs. No enhancement occurred following pairing of both continuous representation and discontinuous representation pathways. (c) Change in PSP amplitude for the continuous representation pathway plotted against discontinuous representation for each cell that received APV and both pathway pairing. Solid diagonal line depicts slope of one. Dashed lines depict 15% above and below no change.

In order to test if the synaptic plasticity induced here was NMDAR dependent, we bath applied a well-known NMDAR antagonist, DL-2-amino-5-phosphonovaleric acid (APV). In a subset of neurons (7 cells) pairing was performed on both pathways in the presence of APV (100 *μ*M). APV blocked all plasticity (Figure 5). Thus, the enhancements and depressions observed via the pairing paradigm were NMDA receptor-dependent.

### 3.6. Horizontal Connections Are Plastic after Robust Depolarization of the Postsynaptic Target

**Figure 6:**
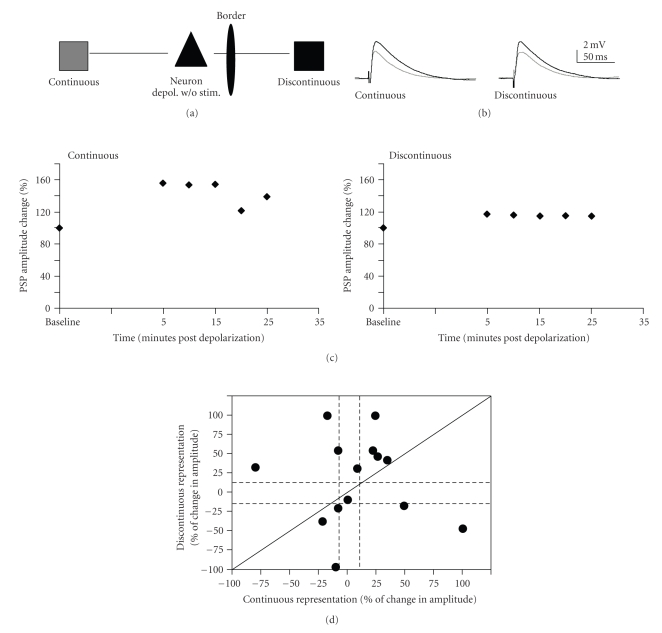
LTP of neurons that received depolarization without synaptic stimulation. (a) Schematic of connections. No pairing was given, only robust depolarization. (b) Example of PSPs. Gray traces are averaged baseline PSPs. Black traces are averaged traces 20 minutes postpairing. Traces are averaged over 5 minutes (15 traces) for both pre- and postpairing PSPs. (c) % change of PSP amplitude over time for the cell shown in 2. PSP amplitude change was averaged over five minute intervals. (d). Individual data: change in PSP amplitude (expressed as percent of the baseline PSP amplitude) for continuous representation plotted against discontinuous representation pathway for each cell that received depolarization only. Conventions are as in Figure 4.

In order to examine possible nonspecific effects of depolarization on inputs, cells (14 cells in 9 animals) were subject to the depolarization paradigm (200-milliseconds of current to yield ~10 action potentials, repeated 30 times), but the inputs to the cell were not stimulated during the depolarizations (Figure 6). Twenty minutes after this series of depolarizations, the PSPs evoked by discontinuous and continuous pathway stimulation did not significantly differ from baseline, as measured by mean percent change in amplitude (One-sample *t*-test; *P* = .09). Although the pattern of changes for individual cases varied (Figure 6(d), Table 2), the plasticity outcome induced just by depolarization was significantly different from those obtained by pairing of the continuous pathway or both pathways (chi-square: depolarization versus discontinuous *P* = .77; depolarization versus continuous *P* = .026; depolarization versus both paired *P* < .0001). APV (100 *μ*M) was bath applied to determine if this enhancement was mediated through NMDA receptors. APV did not block the effects of depolarization on the PSPs (*N* = 5; Table 2). Thus, enhancements observed via the depolarization paradigm are not dependent on similar mechanisms to those of the pairing paradigm.

### 3.7. LTD of Horizontal Connections

We also examined the ability to induce LTD in these horizontal connections. LFS, a standard way of inducing LTD in neural circuits, was presented to one of the two pathways for a subset of cells. Presentation of LFS to both pathways was never performed as the length of time needed to deliver LFS would result in washout of the cell and plasticity would likely not occur [45]. Additionally, we did not want to stagger the stimulation between the two pathways and create a possible additive effect. 

**Figure 7:**
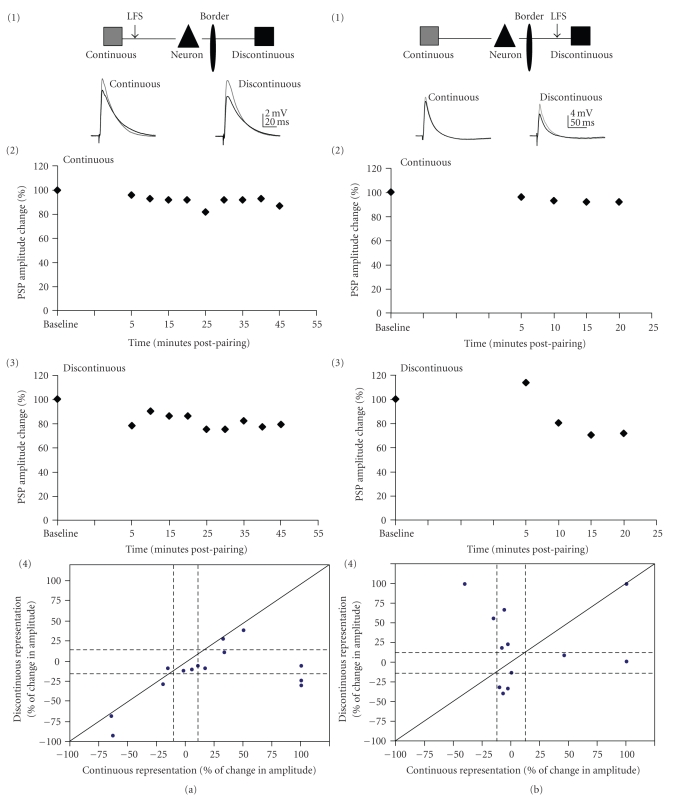
LFS induced plasticity. (a) Fourteen neurons underwent LFS to the continuous representation connections only. (b) Twelve neurons underwent LFS of discontinuous representation connections only. (1) Schematic drawing of the in vitro preparation. (2) Example responses from sample cells. Traces are averaged over five minutes with grey showing PSPs from before pairing (baseline) and black showing those from 20 minutes postpairing. (3): % change of PSP amplitude over time for the cell shown in (2). PSP amplitude change was averaged over five minute intervals. (4) Change in PSP amplitude (expressed as percent of the baseline PSP amplitude) for continuous representation pathway plotted against discontinuous representation pathway for each cell. Solid diagonal line depicts slope of one. Dashed lines depict 15% above and below no change (100%).

Figure 7 shows the results of LFS to the continuous pathway (*N* = 14; Figure 7(a)) and to the discontinuous (*N* = 12; Figure 7(b)). The mean amplitudes of PSPs from either pathway showed no significant changes after either LFS paradigm (Wilcoxon-sign test: discontinuous LFS *P* = .95; continuous LFS *P* = .61; Figure 7). As demonstrated for pairing, however, individual neurons exhibited a variety of plasticity outcomes in response to LFS (4 in Figures 7(a) and 7(b); Table 3(b)). These outcomes were significantly different from each other and also different from the outcomes resulting from pairing (chi-square test: *P* = .006). Like the results from pairing, LFS of the discontinuous pathway was more likely (100% of cases) to cause a long-lasting change in synaptic efficacy, either LTD or LTP, than LFS to the continuous (78.6% of cases; Table 3). The likelihood of LTD and LTP was approximately equal when the LFS was presented. 

Unlike the results for pairing, the continuous and the discontinuous pathway underwent LTD and LTP with approximately equal probability after LFS (Table 3(b)). Heterosynaptic LTD could be observed in approximately equal numbers of cases (14.5% for continuous-pathway LFS and 16.7% for discontinuous pathway LFS; Table 3(b)). However, continuous-pathway LFS induced no heterosynaptic LTP (0%), while discontinuous-pathway LFS did exhibit heterosynaptic LTP (16.7%). Thus, as for pairing-induced plasticity, the discontinuous pathway was less able to affect the continuous than vice versa.

In order to determine if the depression observed in these horizontal pathways was typical NMDAR-dependent LTD, we bath applied APV (100 *μ*M; *N* = 6) while presenting LFS to the continuous representation pathway (Table 3(b)). Long-lasting changes in synaptic amplitude were still observed in 83.3% of cells: in 33.3% of cases both pathways depressed and in 50% of cases both pathways enhanced. Hence, the synaptic plasticity evoked by LFS was not NMDA receptor dependent. The pattern of change was not significantly altered in the presence of APV (chi-square test LFS APV versus discontinuous LFS *P* = .6; chi-square test LFS APV versus continuous LFS *P* = .4), although there was a notable enhancement of LTP as an outcome and a reduction in pathway-specific effects.

## 4. Discussion

One-way circuits in the brain can undergo change is via LTP and LTD. Both have been linked to learning and memory [46–48] and have been proposed to underlie circuit changes following sensory loss and remapping of function in the brain [20]. The experiments detailed here demonstrate that the horizontal connections in somatosensory cortex are plastic, even in the adult, and that the functional organization of the cortex may influence the susceptibility of connections to synaptic plasticity. 

### 4.1. Pairing-Induced Synaptic Plasticity

We located the forepaw/lower jaw border in vivo and then probed the circuitry surrounding this border in vitro. The PSPs elicited by horizontal stimulation were similar to those previously recorded [10, 16]. Previous control studies from our lab demonstrate that the circuitry is not altered due to in vivo mapping or the marking of the border [10, 12]. The pairing paradigm used in these experiments is not the same pairing paradigm used in spike time dependent plasticity since the cell is depolarized to a point where it fires 7–10 action potentials for the duration of the 200-millisecond depolarization. The pairing paradigm has been extensively studied and usually yields potentiation in juvenile cortex and the hippocampus [14, 21, 25, 32]. When examined as a population, there was little apparent long-lasting synaptic change induced by this paradigm. However, examination of individual neurons revealed significant and diverse forms of synaptic plasticity for both continuous and discontinuous pathways (Table 2). We report that the pairing paradigm yielded all three forms of synaptic plasticity when pairing was presented to the discontinuous representation pathway: associative (both pathways paired, both enhance), homosynaptic (discontinuous paired and enhances) and heterosynaptic (discontinuous paired, continuous enhances). However, when continuous representation pathways was presented with plasticity paradigms, only homosynaptic plasticity (continuous paired and enhances) and associative plasticity (continuous paired and both enhance) were observed (Figures 2(a) and 4). In general connections that crossed map discontinuities were less likely to undergo plasticity when adjacent horizontal connections received changes in activity (Table 2). Furthermore, when the discontinuous pathway did potentiate, it was a result of homosynaptic plasticity (i.e., when pairing was provided to the discontinuous pathway); by contrast, pairing of the continuous pathway also exhibited heterosynaptic effects (i.e., enhancement of the discontinuous pathway; Table 2). Clearly, continuous pathway stimulation was able to engage pairing-induced synaptic plasticity to a greater extent than discontinuous. For both pathways, the potentiation observed was dependent on NMDAR activation, as has been reported previously [49].

This pattern of plasticity is consistent with our previous data that the discontinuous pathway is less able to excite its targets than the continuous [10], because it provides fewer axons to its targets [9] and its individual excitatory synapses are weaker than those of the continuous [43]. In a Hebbian system, weaker pathways onto a target are less able to induce homosynaptic plasticity but can be potentiated by association with concurrent plasticity in stronger inputs [50].

### 4.2. LFS-Induced Synaptic Plasticity

The results from LFS stimulation of the two pathways showed significant similarities and differences from the results from pairing. For LFS stimulation, discontinuous pathway LFS was more able to induce synaptic plasticity in either direction than continuous pathway LFS (Table 3(b)). However, LFS to the continuous pathway was more likely to affect the discontinuous pathway (i.e., heterosynaptic plasticity) than vice versa (Table 3(b)), which was similar to the results of pairing. The synaptic plasticity induced by LFS was not dependent on NMDAR activation, which is different than previously reported for cortex [49]. However, blocking NMDAR did alter the characteristics of synaptic plasticity induced by LFS; in particular it increased the likelihood that the LFS paradigm would result in enhancement of the responses to either pathway (Table 3(b)). One possible explanation may be the LTD induced here is dependent on endocannabinoid signaling which has been reported in young rats [51]. Additionally, homosynaptic NMDAR-independent LTD has also been observed previously in the neocortex [52].

### 4.3. Interactions between LTP and LTD

We also found unexpected plasticity outcomes in these pathways; the pairing paradigm, which typically generates LTP in other systems, induced depression in a subset of cells in this study, while the LFS paradigm induced potentiation in some cells. Unexpected plasticity outcomes were observed in both continuous and discontinuous pathway synapses (Tables 2 and Figure 2(b)). Similar results have previously been observed in cortex in several contexts. As mentioned above, LTP induction in one set of inputs can lead to endocannabinoid-dependent LTD in other pathways [51]. Furthermore, in young rats, a pairing paradigm presented to connected pairs of layer II/III pyramidal neurons can yield enhancement, depression, or no change in the EPSP amplitude, depending upon the probability of release for that synapse and its location on the target. Furthermore, connections with low release probabilities and those made onto more distal dendrites were more likely to enhance [53]. Thus, it is clear that pairing protocols can yield both homosynaptic and heterosynaptic depression in young rats. Another factor that controls the direction of synaptic plasticity is the size of the calcium signal in the target cell. In cortex, small rises in intracellular Ca^2+^ are associated with LTD induction, while larger rises produce LTP [54]. Although in hippocampus no such relationship was found [55]. Our data indicate that similar processes can occur in adult rat cortex, although the precise mechanisms underlying them remain to be determined.

Unexpected plasticity outcomes may also be a result of the complex horizontal cortical circuitry in superficial cortex. The vast majority of what we know about LTP and LTD is derived from studies examining hippocampal LTP [24, 25, 56]. In hippocampal slices, the PSPs that are enhanced are monosynaptic [57]. Furthermore, in many studies inhibition is blocked. In contrast, PSPs examined here are more complex and are derived from monosynaptic and polysynaptic activity, as well as having both excitatory and inhibitory components [10]. It is important to note that inhibitory synapses were not blocked during any of the recordings in this study. Therefore, any enhancement or depression seen in these experiments was in the presence of intact excitation and inhibition. This way plasticity was induced in conditions similar to in vivo conditions. It has been demonstrated that inhibitory synapses are susceptible to both LTP and LTD based on a variety of induction protocols, including LFS, tetanus, and pairing. Our results are consistent with those of other laboratories using a pairing paradigm in adult cortex where inhibition was not blocked [58, 59]. Thus, some of the diversity of plasticity outcomes that we observed may be explained by the diverse connection patterns of single layer II/III neurons, the balance of excitation and inhibition, and the amplitude and time course of the calcium transients present in the neuron during various synaptic plasticity induction protocols.

### 4.4. NonHebbian Synaptic Plasticity

NonHebbian synaptic plasticity was observed within these connections. We observed enhancement following robust depolarization without any pairing with synaptic stimulation (Figure 6; Table 2). This enhancement was not NMDA receptor dependent and thus operated via different mechanisms than the LTP observed following pairing. Other studies have shown similar phenomena of long lasting enhancement or LTP induced by long depolarizing steps with no presynaptic stimulation in both the cortex [60] and hippocampus [61]. Evidence suggests that this type of depolarization-induced enhancement is due to calcium influx from depolarization [62], which is most probably mediated through voltage-dependent calcium channels (VDCCs) [63]. Data using photolysis of caged Ca^2+^ in hippocampal neurons directly demonstrated that this process depends on intracellular Ca^2+^  and that it can induce either LTP or LTD of synapses onto the target neurons [55].

### 4.5. Estrogen and Plasticity

One potential source for the variability in plasticity outcome for individual cells is the use of only female rats of unknown estrous state in these studies. It has been demonstrated that female rats change in their susceptibility to LTP and LTD during the estrous cycle. In particular, during proestrous when estrogen levels are high, the ability to induce LTP was enhanced and the ability to induce LTD was depressed in the hippocampus [64, 65]. It has been hypothesized that the effects on synaptic plasticity are due to the modulation of NMDA or GABA receptors or by an increase in dendritic spine density induced by estrogen [64]. Since the rats in our studies came from random points in their estrous cycles, some would be in this high-estrogen state. However, proestrus is short in the rat, lasting less than 18 hours, which makes it approximately 15% of the entire cycle [66]. Thus, only about 15% of our rats would show this effect, which is insufficient to explain the observed variance. This effect on LTP and LTD has not explicitly been demonstrated in the neocortex, although the increase in spine density during proestrous has been observed in both hippocampus and neocortex [67]. Furthermore, the mechanism by which estrogen modulates synaptic plasticity is also an important issue. For example, if estrogen modulates LTP and LTD via NMDA or GABA receptors, the effect would not be observed in our in vitro system since any acute estrogen effect would be washed out; if the modulation depends on the increase in spines, this would still be observed in the slices used. Overall, given the short period of time that the animals are in proestrus, and the use of cortical slices for this study, we do not believe that the differences observed in synaptic plasticity can be accounted for by the use of female rats. 

In summary, the present study demonstrates that synapses of intracortical connections of S1 can undergo synaptic plasticity. Furthermore, the presence of a representational border affects the capacity of these synapses to undergo plasticity. In general, connections from an adjacent representation are less able to undergo plasticity on their own. Nonassociative synaptic plasticity was also observed following robust depolarization of layer II/III neurons. These results suggest that synapses originating from the different representations have different characteristics, for example, various locations of synapses on postsynaptic targets. Additionally, the results also suggest that there are multiple forms and loci (i.e., excitatory and inhibitory synapses) of synaptic plasticity that affect the expression of plasticity in a complex circuit, such as supragranular S1. While the plasticity that was seen here was diffuse and not all neurons responded to the plasticity paradigms uniformly, large-scale alterations of activity due to deafferentation would still have the capacity to alter the circuit. The net result would depend on the specific interaction of these varied plasticity mechanisms in layer II/III with processes occurring in other layers and the extent of the change in activity. The data here indicate that LTP and/or LTD have the potential to play roles in cortical reorganization following sensory loss.

## Figures and Tables

**Table 1:** Cell parameters for LTP.

	*N*	RMP (mV)	Input resistance (MW)	Distance (border) (*μ*m)	Distance (pia) (*μ*m)
Both paired	25	−75.2 ± 0.9	93.8 ± 8.1	115.8 ± 8.4	329.0 ± 10
Continuous representation pathway paired	16	−73.5 ± 1.1	101.0 ± 7.4	103.1 ± 7.1	289.0 ± 12
Discontinuous representation pathway paired	14	−78.3 ± 0.9	116.0 ± 9.1	103.9 ± 4.4	321.4 ± 9
Both paired + APV	7	−77.0 ± 2.4	119.7 ± 24.5	100.0 ± 0.0	287.5 ± 10
Depolarization	14	−72.2 ± 1.1	136.7 ± 13.9	93.2 ± 9.8	306.7 ± 7
Depolarization + APV	5	−75.6 ± 2.6	102.0 ± 19.7	80.0 ± 10.5	300.0 ± 0

**Table 2:** Summary of results for LTP changes in amplitude (% occurring for each group).

	Continuous representation pathway enhances	Discontinuous representation pathway enhances	Both enhance	No change	Both Depress
Both paired	32.0	0.0	12.0	28.0	28.0
Continuous pathway paired	25.0	0.0	37.5	0.0	37.5
Discontinuous pathway paired	28.6	21.4	28.6	0.0	21.4
Both paired + APV	0.0	0.0	0.0	100.0	0.0
Depolarization	14.3	28.6	28.6	7.1	21.4
Depolarization + APV	20.0	40.0	20.0	0.0	20.0

**Table:** (a) Cell parameters for LFS.

	*N*	RMP	Input Resistance	Distance (border)	Distance (pia)
		(mV)	(MΩ)	(*μ*m)	(*μ*m)
Continuous representation pathway LFS	14	−74.9 ± 0.9	111.9 ± 10	105.3 ± 9.5	303.5 ± 16.1
Discontinuous representation pathway LFS	12	−74.0 ± 0.8	103.2 ± 11.6	116.7 ± 11.7	283.3 ± 11.2
Continuous representation pathway LFS+APV	6	−73.8 ± 1.6	95 ± 17.0	103.5 ± 3.3	325.0 ± 17.0
Picrotoxin	3	−76.0 ± 3.5	74.6 ± 1.3	75.0 ± 14.4	350.0 ± 0.0

**Table:** (b) Summary of Results for LTD. Changes in Amplitude (% occurring for each group).

	Cont. depress	Discont. Depress	Both depress	No change	Cont. enhance	Discont. enhance	Both Enhance
Continuous pathway LFS	7.1	14.3	21.4	21.4	21.4	0.0	14.3
Discontinuous pathway LFS	16.7	33.3	0.0	0.0	16.7	25.0	8.3
Continuous pathway LFS + APV	0.0	0.0	33.3	16.7	0.0	0.0	50.0
